# LncRNA CTD-2528L19.6 prevents the progression of IPF by alleviating fibroblast activation

**DOI:** 10.1038/s41419-021-03884-5

**Published:** 2021-06-10

**Authors:** Tingting Chen, Yingying Guo, Jiayi Wang, Liqiang Ai, Lu Ma, Wenxin He, Zhixin Li, Xiaojiang Yu, Jinrui Li, Xingxing Fan, Yunyan Gu, Haihai Liang

**Affiliations:** 1 College of Bioinformatics Science and Technology, Harbin Medical University, Harbin, China; 2 Department of Pharmacology (State-Province Key Laboratories of Biomedicine-Pharmaceutics of China, Key Laboratory of Cardiovascular Research, Ministry of Education), College of Pharmacy, Harbin Medical University, Harbin, China; 3 Northern Translational Medicine Research and Cooperation Center, Heilongjiang Academy of Medical Sciences, Harbin Medical University, Harbin, China; 4 Department of Thoracic Surgery, Shanghai Pulmonary Hospital, Tongji University, Shanghai, China; 5 State Key Laboratory of Quality Research in Chinese Medicine/Macau Institute for Applied Research in Medicine and Health, Macau University of Science and Technology, Macau, China; 6 Research Unit of Noninfectious Chronic Diseases in Frigid Zone (2019RU070), Chinese Academy of Medical Sciences, Harbin, China

**Keywords:** Mechanisms of disease, Non-coding RNAs

## Abstract

Long non-coding RNAs (lncRNAs) have emerged as critical factors for regulating multiple biological processes during organ fibrosis. However, the mechanism of lncRNAs in idiopathic pulmonary fibrosis (IPF) remains incompletely understood. In the present study, two sets of lncRNAs were defined: IPF pathogenic lncRNAs and IPF progression lncRNAs. IPF pathogenic and progression lncRNAs-mRNAs co-expression networks were constructed to identify essential lncRNAs. Network analysis revealed a key lncRNA *CTD-2528L19.6*, which was up-regulated in early-stage IPF compared to normal lung tissue, and subsequently down-regulated during advanced-stage IPF. *CTD-2528L19.6* was indicated to regulate fibroblast activation in IPF progression by mediating the expression of fibrosis related genes *LRRC8C, DDIT4, THBS1, S100A8* and *TLR7* et al. Further studies showed that silencing of *CTD-2528L19.6* increases the expression of Fn1 and Collagen I both at mRNA and protein levels, promoted the transition of fibroblasts into myofibroblasts and accelerated the migration and proliferation of MRC-5 cells. In contrast, *CTD-2528L19.6* overexpression alleviated fibroblast activation in MRC-5 cells induced by TGF-β1. LncRNA *CTD-2528L19.6* inhibited fibroblast activation through regulating the expression of *LRRC8C* in vitro assays. Our results suggest that *CTD-2528L19.6* may prevent the progression of IPF from early-stage and alleviate fibroblast activation during the advanced-stage of IPF. Thus, exploring the regulatory effect of lncRNA *CTD-2528L19.6* may provide new sights for the prevention and treatment of IPF.

## Introduction

Idiopathic Pulmonary Fibrosis (IPF) is a progressive and chronic disorder that has been characterized by excessive wound repair and fibrosing interstitial pneumonia of unknown etiology^1,2^. IPF is likely driven by abnormal epithelium and propagated by dysregulated overabundant, heterogeneous fibroblast population in various states of activation^3–5^. It has been reported that transforming growth factor-β1 (TGF-β1) plays a key role in the development of IPF and TGF-β1 gene polymorphisms may affect disease progression in patients with IPF^6^. Survival rate of the advanced-stage IPF patients at 5 years is much lower than that of early-stage IPF^7^. Although emerging evidence indicates that genetic studies may hold promise in the connections between early-stage and advanced-stage disease^8^, no special study focuses on analyzing the difference of gene expression during different stages of IPF. Thus, a comprehensive understanding of the pathogenesis and progression mechanisms involved in IPF remains elusive.

Long non-coding RNAs (lncRNAs) are one type of non-coding RNAs with lengths greater than 200 nucleotides. The aberrant expression of lncRNAs has been linked to multiple biological processes involved in IPF^9–11^. Some studies developed IPF signatures based on lncRNAs expression analysis. For example, lncRNA *H19* functions as a molecular sponge of *miR-196a*, which provides a novel therapeutic target for IPF^12–14^. LncRNA *MALAT1*, *E2F1*, and *YBX1* may be key regulators for the pathogenesis of IPF, in the peripheral blood of IPF patients^15^. LncRNA *sirt1* antisense was reported to inhibit TGF-β1-mediated epithelial-mesenchymal transition in vitro and alleviate the progression of IPF in vivo^16^. Crosstalk among non-coding RNAs plays a crucial regulatory role in the progression of IPF^17^. Savary et al. demonstrated that lncRNA *DNM3OS* regulates myofibroblast activation by giving rise to profibrotic mature miRNAs, such as *miR-199a-5p/3p* and *miR-214-3p*^18^. Interfering with *DNM3OS* function not only prevents lung fibrosis but also improves established pulmonary fibrosis. LncRNA *ZFAS1* promotes the progression of pulmonary fibrosis and facilitates the proliferation and phenotypic differentiation of fibroblasts into myofibroblasts via functioning as a ceRNA^19^, and lncRNA *Hoxaas3* promotes lung fibroblast activation and fibrosis by targeting *miR-450b-5p* to regulate *Runx1*^20^. Huang et al. demonstrated that lncRNA *FENDRR* exhibits anti-fibrotic activity in pulmonary fibrosis^21^. Recently, we have reported several functional lncRNAs as targets for the treatment of fibrosis, and designated three pulmonary fibrosis regulatory associated lncRNAs (*PFRL, PFAL, PFAR*) that promote lung fibrosis by competitively binding miRNA^22–24^.

Previous studies provided insights into the crosstalk between mRNAs and lncRNAs to explore the regulatory mechanism in pulmonary fibrosis^25,26^. The changes in lncRNAs expression may affect the stability and translation of genes involved in lung fibrosis^26^. Hao et al. reported that lncRNA *AP003419.16* regulates its adjacent gene *RPS6KB2*, which regulates the process of IPF^27^. Increasing studies suggest new theories for the pathogenesis and treatments of IPF^25^. Thus, systematically exploring the deregulation mechanism between lncRNAs and mRNAs in different stages of IPF will enhance our understanding of the progression of IPF.

In this study, we identified IPF pathogenic and progression related mRNAs and lncRNAs by constructing a pathogenic network and two dynamic progression networks for IPF. Our study revealed a core IPF regulatory sub-network centered on the lncRNA *CTD*-*2528L19*.6, which was up-regulated in the early-stage of IPF patients but down-regulated in advanced-stage IPF patients. In vitro assays, consistent with bioinformatics analysis of IPF progression in context: *CTD-2528L19.6* was proven to alleviate fibroblast activation by regulating *LRRC8C* in MRC-5 cells and the silencing *CTD-2528L19.6* promoted pulmonary fibrosis. In summary, our study highlighted that *CTD-2528L19.6* may prevent the progression of IPF by alleviating fibroblast activation.

## Results

### Identification of IPF pathogenic signature and IPF progression signature

**Fig. 1:**
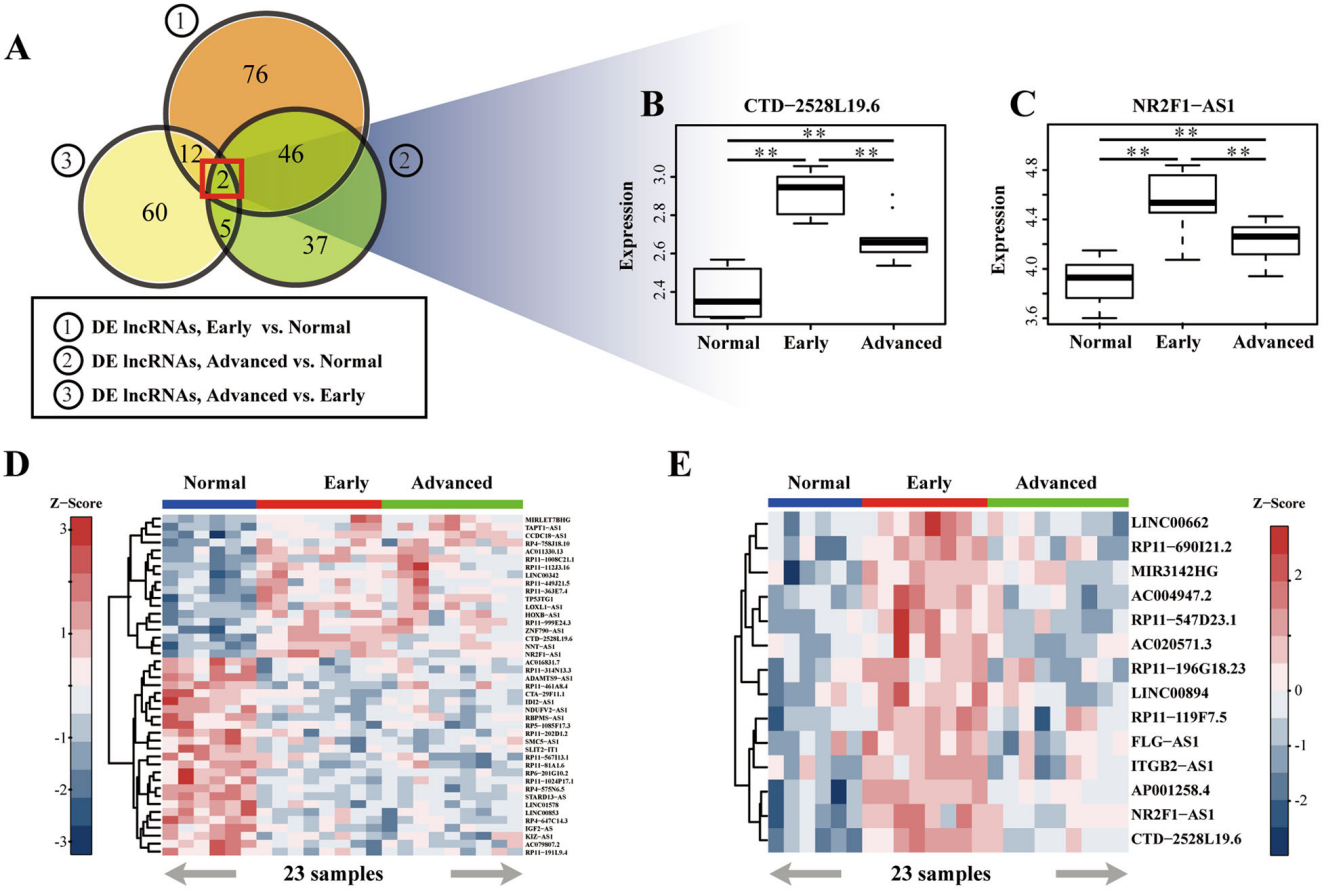
Identification of IPF lncRNAs signatures. **A** Venn diagram of overlapping DE lncRNAs among three groups. **B**, **C** Distribution of expression levels for key lncRNAs *CTD*-*2528L19*.6 and *NR2F1-AS1*. *P < 0.05, **P < 0.01 in Student’s *t*-test. **D**, **E** Heatmaps show the global expression levels of IPF pathogenic lncRNAs (**D**) and IPF progression lncRNAs (**E**) in GSE24206.

To evaluate gene expression patterns in the occurrence and development of IPF, we identified differentially expressed (DE) genes (DEGs) for the following three comparisons: (1) early-stage IPF *vs*. normal; (2) advanced-stage IPF *vs*. normal; (3) advanced-stage IPF *vs*. early-stage IPF. The methodological workflow for this study is summarized in Supplementary Fig. S1. Two lncRNAs and 48 mRNAs were significantly differentially expressed in all three comparisons (*P* < 0.01, Student’s *t*-test, Fig. 1A; Supplementary Fig. S2A). LncRNA *CTD*-*2528L19.6*, *NR2F1*-*AS1* were significantly up-regulated in early-stage of IPF compared with normal lung and then down-regulated in advanced-stage of IPF (*P* < 0.01, Student’s *t*-test, Fig. 1B, C). Notably, the expression level of lncRNAs *CTD*-*2528L19.6* and *NR2F1*-*AS1* was still higher in advanced-stage IPF than that in normal lung (*P* < 0.01, Student’s *t*-test, Fig. 1B, C). Also, some genes, such as *DCLRE1C*, *S100A8*, *THBS1*, showed reverse tendency among the normal, early-stage and advanced-stage of IPF (*P* < 0.01, Student’s *t*-test, Supplementary Fig. S2B-D).

Then, 43 DE lncRNAs and 835 DE mRNAs that were differentially expressed in both early-stage and advanced-stage IPF compared to normal samples, were defined as IPF pathogenic signatures. IPF pathogenic lncRNAs and mRNAs showed consistently up-regulated or down-regulated expression in both early-stage and advanced-stage IPF compared with normal samples (Fig. 1D for lncRNAs, Supplementary Fig. S2E for mRNAs). 14 DE lncRNAs and 264 DE mRNAs that were differentially expressed in advanced-stage IPF compared to both normal and early-stage samples, were defined as IPF progression signature. Interestingly, all IPF progression lncRNAs were sharply up-regulated in the transition from normal lung to early-stage IPF, then there was a “rebound” tendency. The expression level of IPF progression lncRNAs was obviously down-regulated during the transition to advanced-stage IPF, but was still higher compared to normal lung (Fig. 1E). A similar “rebound” tendency was observed in IPF progression mRNAs (Supplementary Fig. S2F).

Additionally, we performed pathway enrichment analyses to investigate the potential functional mechanisms involved in pathogenesis and progression of IPF. IPF pathogenic mRNAs participated in pathways related to immune system and inflammation, including “Phagosome”, “Antigen processing and presentation”, “Cell adhesion molecules (CAMs)”, “Focal adhesion” and “ECM-receptor interaction” (*FDR* < 0.05, Hypergeometric test, Supplementary Fig. S2G). IPF progression mRNAs are involved in some IPF-related pathways, including “PI3K/Akt signaling” and immune system such as “B cell receptor signaling pathway”, “Chemokine signaling pathway” and “Jak-STAT signaling pathway” (*FDR* < 0.05, Hypergeometric test, Supplementary Fig. S2H). The above results indicated that dysregulation of lung fibrosis and immune systems plays important roles in IPF^28,29^.

### Analysis of IPF pathogenic co-expression network

**Fig. 2:**
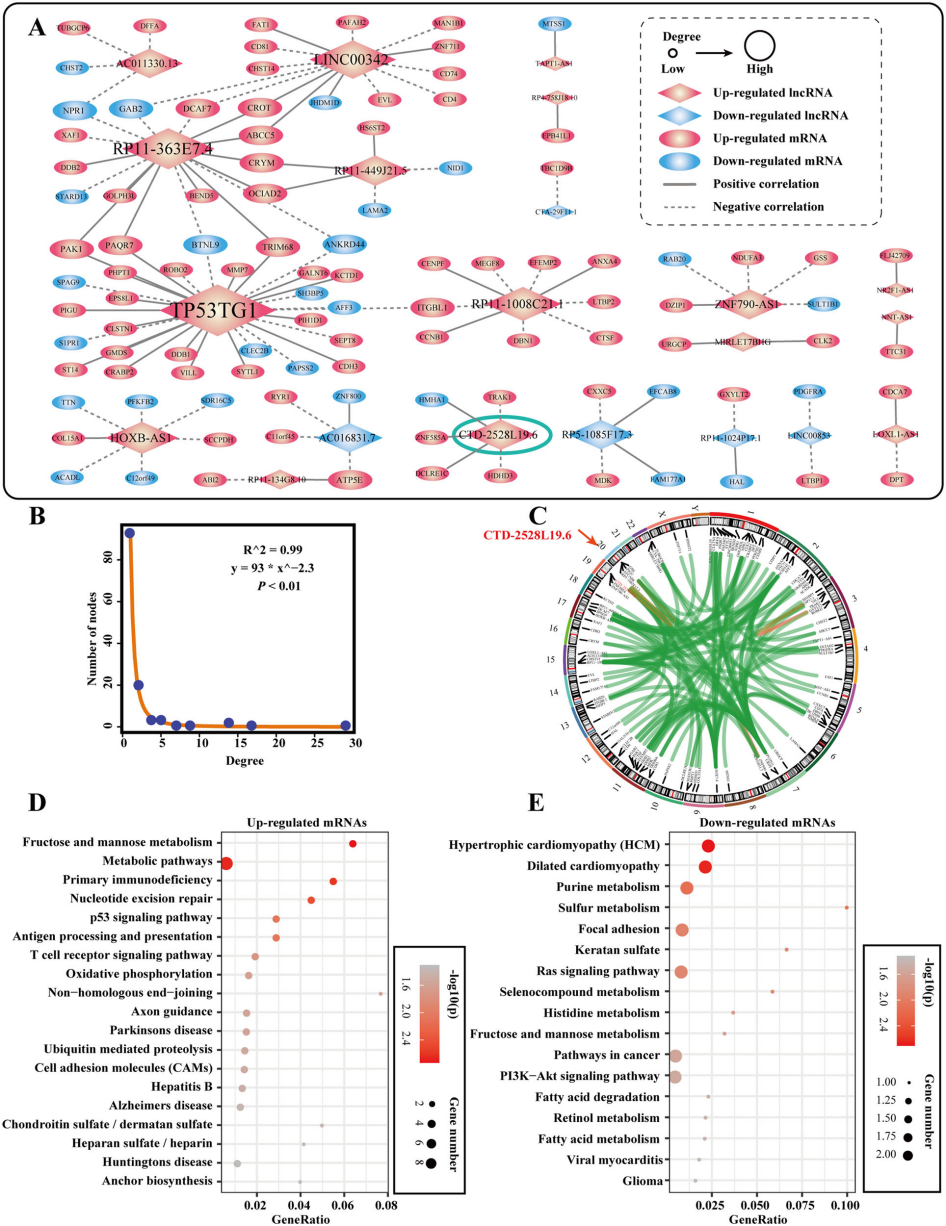
LncRNA-mRNA co-expression networks during the pathogenesis of IPF. **A** IPF pathogenic lncRNAs (diamond)-mRNAs (oval) co-expression network in IPF. The nodes marked with red color represent the up-regulated IPF pathogenic genes. The nodes marked with blue color represent the down-regulated IPF pathogenic genes. The solid lines (dotted lines) represent the positive (negative) correlation between mRNAs and lncRNAs (|r| >0.8, *P* < 0.01, *P*earson Correlation Test). **B** Distribution of the degree of genes in the IPF pathogenic network. **C** Circos plots display chromosomal interactions between co-expressed lncRNAs and mRNAs. The location of lncRNA *CTD*-*2528L19*.6 was marked with red arrows. **D**, **E** KEGG pathways enrichment with IPF pathogenic up-regulated mRNAs (**D**) or down-regulated mRNAs (**E**). *FDR* < 0.05, Hypergeometric test. Size of the bubble indicates the number of genes in the corresponding annotation. Color shade corresponds to the -log10(*p*) value. The closer the color is to red, the more significant the enrichment is.

To capture the core lncRNA-mRNA regulatory module over pathogenesis in IPF, we constructed IPF pathogenic co-expression network (Fig. 2A). IPF pathogenic co-expression network exhibited the power-law behavior in “scale-free” network models (*R*^2^ = 0.99, *P* < 0.01, Goodness of fit test) (Fig. 2B). The lncRNAs and mRNAs with co-expression relationships were equally distributed across the genome (Fig. 2C). A sub-network centered on *CTD-2528L19.6* emerged in the IPF pathogenic co-expression network. In the dataset of GSE24206, expression of *CTD-2528L19.6* was increased in early-stage of IPF and decreased in advanced-stage IPF. *CTD*-*2528L19.6* showed a similar differential expression tendency in three independent datasets (GSE10667, SRP10849 and GSE73854, Supplementary Fig. S3A-C). Some hub lncRNAs, such as *LINC00342*, *RP11-1008C21.1*, *RP11-363E7.4* and *TP53TG1*, may regulate fibrogenesis by regulating mRNAs *CD4*, *CCNB1, XAF1* and *PAK1* in fibroblast related gene sets, respectively (Supplementary Fig. S4). Besides, mRNAs up-regulated in the IPF pathogenic network were involved in “Primary immunodeficiency”, “Axon guidance”, and “T cell receptor signaling pathway” (*FDR* < 0.05, Hypergeometric test, Fig. 2D). mRNAs down-regulated in the network were involved in pathways in cancer and cancer-related signaling pathways (*FDR* < 0.05, Hypergeometric test, Fig. 2E).

### Dynamic IPF progression lncRNA-mRNA co-expression networks

**Fig. 3:**
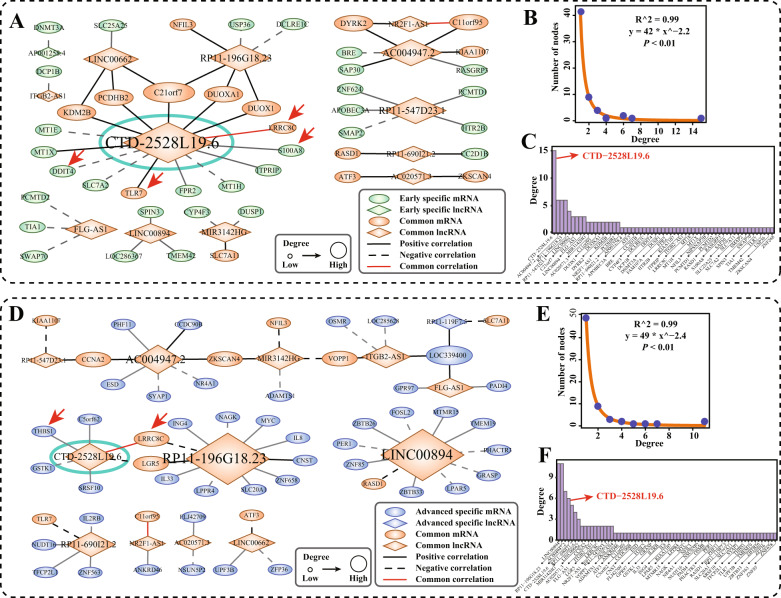
IPF co-expression networks in early-stage and advanced-stage of IPF. **A** IPF progression lncRNAs (diamond)-mRNAs (oval) co-expression network in early-stage IPF. The solid lines (dotted lines) represent the positive (negative) correlation between mRNAs and lncRNAs (|r| >0.8, *P* < 0.01, *P*earson Correlation Test). The nodes marked with green color represent the early-stage IPF specific genes. The nodes marked with yellow color represent the common genes in early-stage and advanced-stage IPF progression network. Several important IPF biomarkers and fibroblast related mRNAs that co-expressed with *CTD-2528L19.6* were marked with red arrows. **B**, **C** Distribution of the degree of genes in early-stage IPF progression network. **D** IPF progression lncRNAs (diamond)-mRNAs (oval) co-expression network in advanced-stage IPF. The nodes marked with blue color represent specific genes in advanced-stage IPF. The nodes marked with yellow color represent the common genes in early-stage and advanced-stage IPF progression network. Several important IPF biomarkers and fibroblast related mRNAs that co-expressed with *CTD-2528L19.6* were marked with red arrows. **E**, **F** Distribution of the degree of genes in the advanced-stage IPF progression network.

**Fig. 4:**
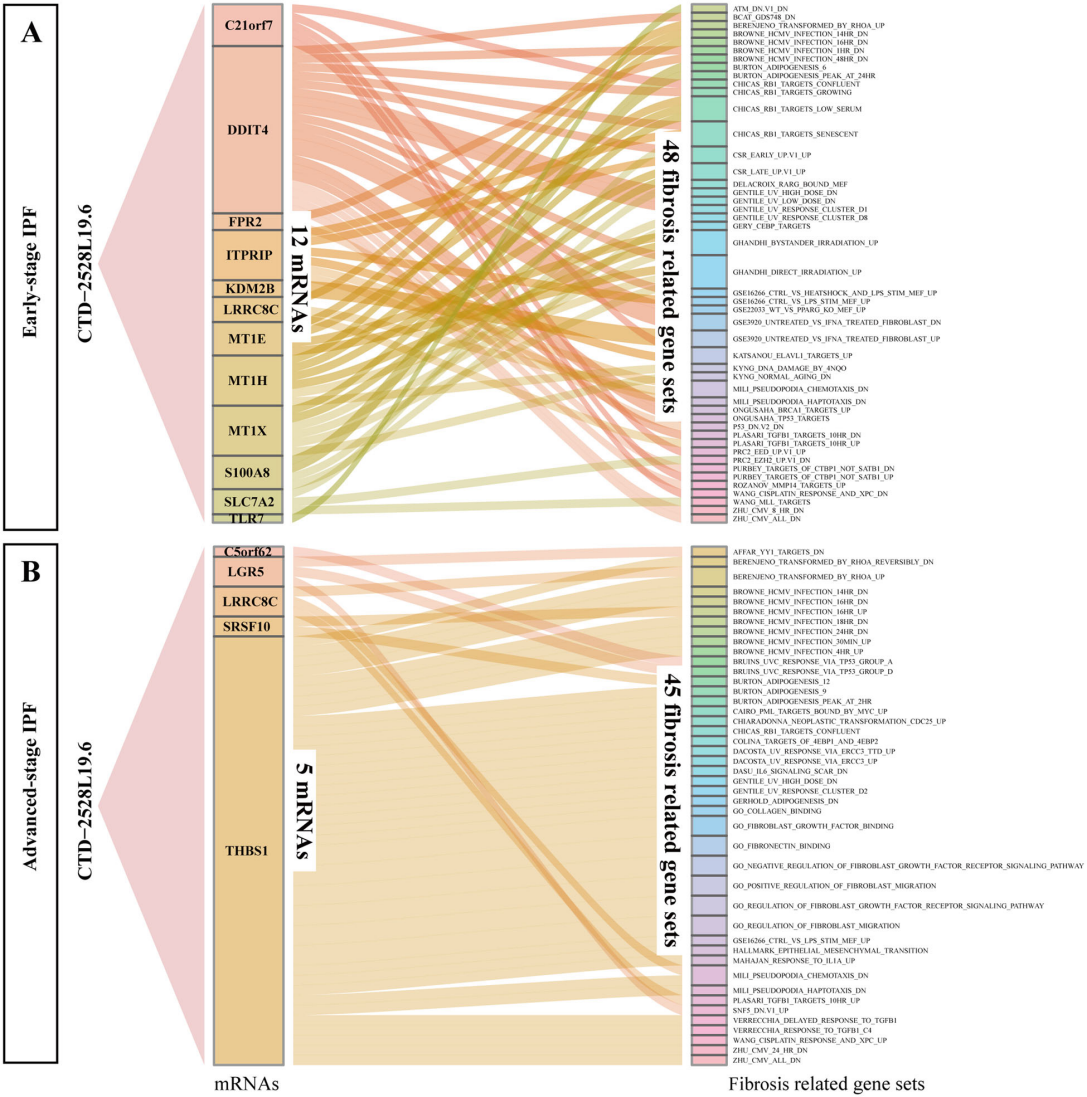
Illustration of fibrosis related gene sets that contain the mRNAs co-expressed with lncRNAs in the progression of IPF. **A, B** Sankey diagrams display the connection between *CTD-2528L19.6* correlated mRNAs and fibrosis related gene sets in early-stage IPF (**A**) and advanced-stage IPF (**B**). The expression of mRNAs in diagrams are correlated with the expression of *CTD-2528L19.6* (|r| >0.8, *P* < 0.01, *P*earson Correlation Test). Path connecting an mRNA to a fibrosis related gene set represents the mRNA participates in the gene set.

To explore the dynamic regulatory mechanism in the IPF progression, IPF early-stage specific and advanced-stage specific lncRNA-mRNA co-expression networks were constructed, respectively. IPF early-stage specific network exhibited the power-law behavior in “scale-free” network models (Fig. 3A; *R*^2^ = 0.99, *P* < 0.01, Goodness of fit test, Fig. 3B), and *CTD-2528L19.6* was co-expressed with 15 mRNAs in this network (Fig. 3C). IPF advanced-stage specific network also exhibited the power-law behavior in “scale-free” network models (Fig. 3D; *R*^2^ = 0.99, *P* < 0.01, Goodness of fit test, Fig. 3E), and *CTD-2528L19.6* was co-expressed with 6 mRNAs in this network (Fig. 3F). *LRRC8C*, which was correlated with *CTD-2528L19.6* in both networks, has been approved to be an IPF biomarker^30^ and is involved in multiple fibrosis related gene sets (Fig. 4A, B). These results emphasized the regulatory relationship between *CTD-2528L19.6* and *LRRC8C* in the dynamic progression of IPF.

mRNAs whose expression correlated with *CTD-2528L19.6* are members of many fibrosis related biological processes. In early-stage IPF co-expression network, mRNAs *S100A8* (*r* = 0.85, *P* = 6.99E-3, Pearson correlation test) and *TLR7* (*r* = 0.89, *P* = 3.01E-3, Pearson correlation test) that co-expressed with *CTD-2528L19.6* have been reported to be novel biomarkers of IPF fibrosis^31,32^. *DDIT4*, whose expression was significantly positively co-expressed with *CTD-2528L19.6* (*r* = 0.87, *P* = 4.24E-3, Pearson correlation test), was contained in 42% of fibrosis related gene sets, which were annotated in the MSigDB database (Fig. 4A). In advanced-stage co-expression network, lncRNAs *RP11-196G18.23*, *LINC00894* and *CTD-2528L19.6* were frequently co-expressed with other genes. IPF biomarker *LRRC8C* was positively co-expressed with *RP11-196G18.23* (*r* = 0.85, *P* = 3.67E-3, Pearson correlation test) and *CTD-2528L19.6* (*r* = 0.81, *P* = 8.71E-3, Pearson correlation test). *THBS1*, which showed significant positive co-expression with *CTD-2528L19.6* (*r* = 0.81, *P* = 8.7E-3, Pearson correlation test), was contained in 84% fibrosis related gene sets (Fig. 4B). These results suggest that *CTD-2528L19.6* affects lung fibrosis in early-stage and advanced-stage of IPF by regulating different fibrosis related genes.

### LncRNA *CTD-2528L19.6* expression is negatively correlated with fibroblast genes

**Fig. 5:**
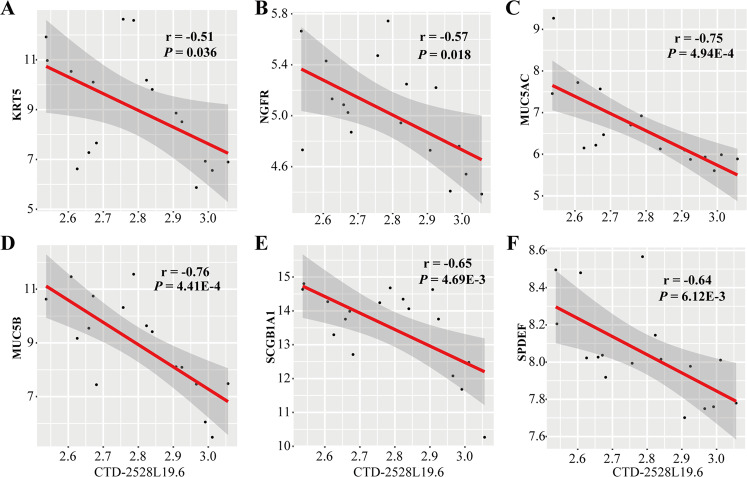
Correlation between *CTD-2528L19.6* and six IPF cell markers in IPF patients. **A**-**F** The correlation relationship between *CTD-2528L19.6* and six IPF cell markers (*KRT5*, *NGFR*, *MUC5AC*, *MUC5B*, *SCGB1A1*, *SPDEF*) was tested by Pearson correlation test. The fitting model was constructed using the “lm” method function in R package ggplot2.

To investigate the association between *CTD-2528L19.6* and fibrosis, we performed Pearson correlation coefficient test between *CTD-2528L19.6* and six IPF cell markers at single-cell level^33^. The results from Fig. 5 showed negative correlations between *CTD-2528L19.6* and IPF cell markers *KRT5* (*r* = −0.51, *P* = 0.036, Fig. 5A), *NGFR* (*r* = −0.57, *P* = 0.018, Fig. 5B), and strong negative correlations between *CTD-2528L19.6* and IPF cell markers *MUC5AC* (*r* = −0.75, *P* = 4.94E-4, Fig. 5C), *MUC5B* (*r* = −0.76, *P* = 4.41E-4, Fig. 5D), *SCGB1A1* (*r* = −0.65, *P* = 4.69E-3, Fig. 5E) and *SPDEF* (*r* = −0.64, *P* = 6.12E-3, Fig. 5F) in IPF patients. Notably, three of the six IPF cell makers (*KRT5*, *NGFR*, *MUC5B*) showed significantly up-regulation in advanced-stage of IPF and the other two makers (*SCGB1A1*, *MUC5AC*) were marginally significantly up-expressed in advanced-stage of IPF compared with early-stage of IPF (Supplementary Fig. S5).

### Silencing lncRNA *CTD-2528L19.6* promotes fibroblast activation in MRC-5 cells

**Fig. 6:**
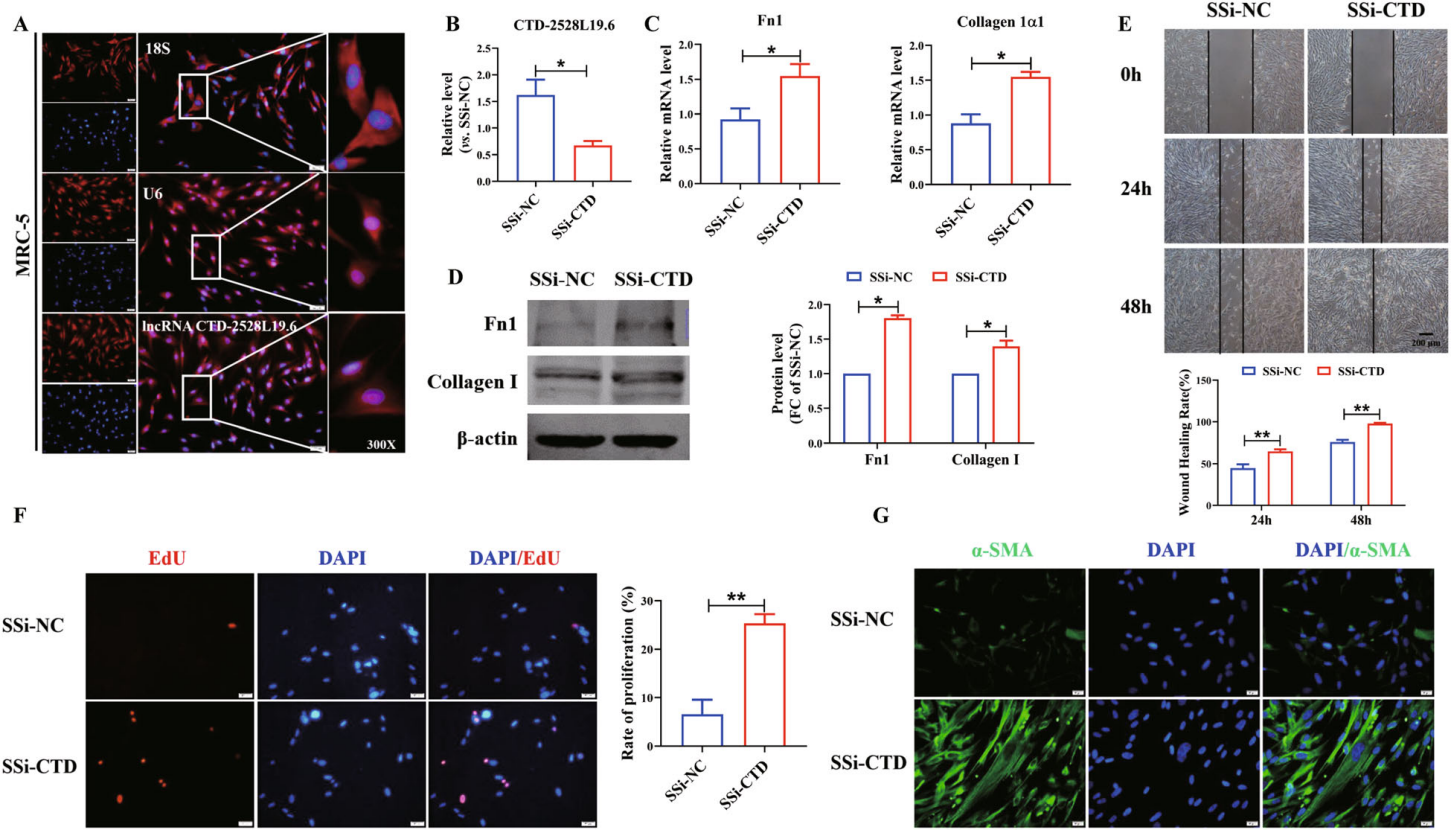
Silencing *CTD*-*2528L19*.6 promotes fibroblast activation of MRC-5 cells. **A** Fluorescence in situ hybridization detects the localization of *CTD*-*2528L19*.6 in MRC-5 cells. For clarity, *CTD*-*2528L19*.6 was abbreviated as *CTD* in the figures. 18 S and U6 are the markers of cytoplasm and nuclei, respectively. Blue nuclei are stained with DAPI. Scale bar: 50 μm. **B**, **C** The expression of *CTD-2528L19.6*, Fn1, Collagen 1α1 after silencing *CTD-2528L19.6* in MRC-5 cells were detected by qRT-PCR. *n* = 4. **D** Western blot was used to detect the expression of Fn1 and Collagen I after SSi-*CTD* transfection. *n* = 6. Wound-healing migration assay (**E**) and EdU proliferation assay (**F**) were used to assay the effect of SSi-*CTD* on migration and proliferation in MRC-5 cells. *n* = 4. Scale bars, 200 μm in **E** and 50 μm in **F**. **G** Immunofluorescence was applied to examine the fibroblasts into myofibroblasts transition after transfecting SSi-*CTD*. Scale bar, 20 μm. *P < 0.05; **P < 0.01.

To determine the effect of lncRNA *CTD-2528L19.6* in lung fibrosis, we first examined the localization of *CTD-2528L19.6* in human lung fibroblast MRC-5 cells by fluorescence in situ hybridization (FISH). FISH results showed that *CTD-2528L19.6* was expressed in both the nucleus and the cytoplasm (Fig. 6A). Then, we used a smart silencer against *CTD-2528L19.6* (SSi-*CTD*) to explore the effects of *CTD-2528L19.6* knockdown on the proliferation, migration and trans-differentiation of MRC-5 cells (Fig. 6B). As illustrated in Fig. 6C, SSi-*CTD* resulted in the up-regulation of Fn1 and Collagen 1α1 at mRNA levels. In addition, silencing *CTD-2528L19.6* promoted the expression of Fn1 and Collagen I at protein levels (Fig. 6D). Meanwhile, as illustrated in Fig. 6E–G, SSi-*CTD* apparently increased the ability of cell migration, proliferation and facilitated the trans-differentiation of fibroblasts into myofibroblasts. The above results suggest that silencing lncRNA *CTD-2528L19.6* can promote the activation of MRC-5 cells.

### Overexpression of *CTD-2528L19.6* alleviates fibroblast activation induced by TGF-β1

**Fig. 7:**
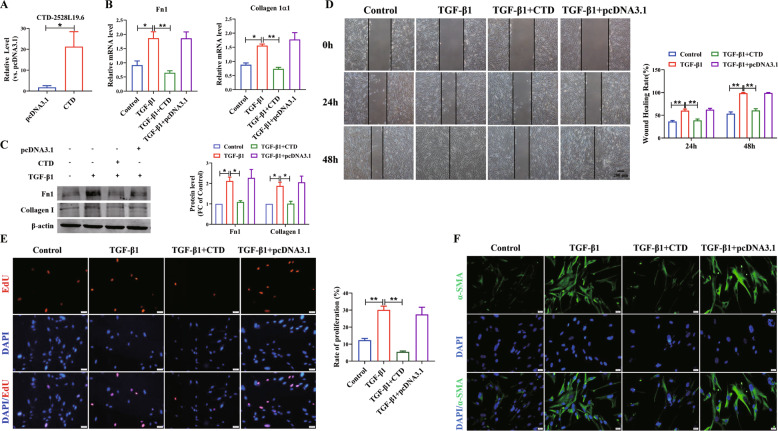
LncRNA *CTD*-*2528L19*.6 inhibits the TGF-β1-induced fibroblast activation in MRC-5 cells. qRT-PCR analysis of the expression of *CTD*-*2528L19*.6 (**A**) and Fn1, Collagen 1α1 (**B**) in MRC-5 cells after transfection *CTD*-*2528L19*.6 under TGF-β1 induced fibroblast activation. *n* = 4. **C** Western blot results demonstrate that the overexpression of *CTD*-*2528L19*.6 inhibits the pro-fibrotic effect of TGF-β1 in MRC-5. *n* = 9. Wound-healing assay (**D**) and EdU proliferation assay (**E**) reveals the inhibitory effects of *CTD*-*2528L19*.6 on TGF-β1-induced migration and proliferation in MRC-5 cells. *n* = 4. Scale bars, 200 μm in **D** and 50 μm in **E**. **F** Fibroblast to myofibroblast transition was observed by immunofluorescence. Scale bar, 20 μm. *P < 0.05; **P < 0.01.

Next, we transfected *CTD-2528L19.6* overexpression plasmid into MRC-5 cells to determine the effect of *CTD-2528L19.6* on fibroblast activation (Fig. 7A). As shown in Fig. 7B, TGF-β1 promoted the expression of Fn1 and Collagen 1α1, which was inhibited by overexpression of *CTD-2528L19.6*. Furthermore, western blot assay showed that forced expression of *CTD-2528L19.6* inhibited the up-regulation of Fn1 and Collagen I induced by TGF-β1 at protein levels (Fig. 7C). Fibrosis occurs with abnormal activation and excessive proliferation of fibroblasts. Wound-healing migration assay showed that overexpression of *CTD-2528L19.6* attenuated TGF-β1-induced cell migration and inhibited wound healing (Fig. 7D). Through the proliferation experiment of the EdU proliferation assay, we found that overexpression of *CTD-2528L19.6* suppressed the MRC-5 proliferation induced by TGF-β1 (Fig. 7E). More importantly, we detected the expression of α-SMA, a marker for fibroblast-myofibroblast transition, in MRC-5 cells by immunofluorescence assay. As shown in Fig. 7F, TGF-β1 could significantly increase α-SMA expression, whereas this effect was inhibited by the overexpression of *CTD-2528L19.6*. These results suggest that overexpression of lncRNA *CTD-2528L19.6* can suppress activation of MRC-5 cells induced by TGF-β1.

### Silencing *LRRC8C* alleviated the inhibitory effect of *CTD-2528L19.6* on fibroblast activation

**Fig. 8:**
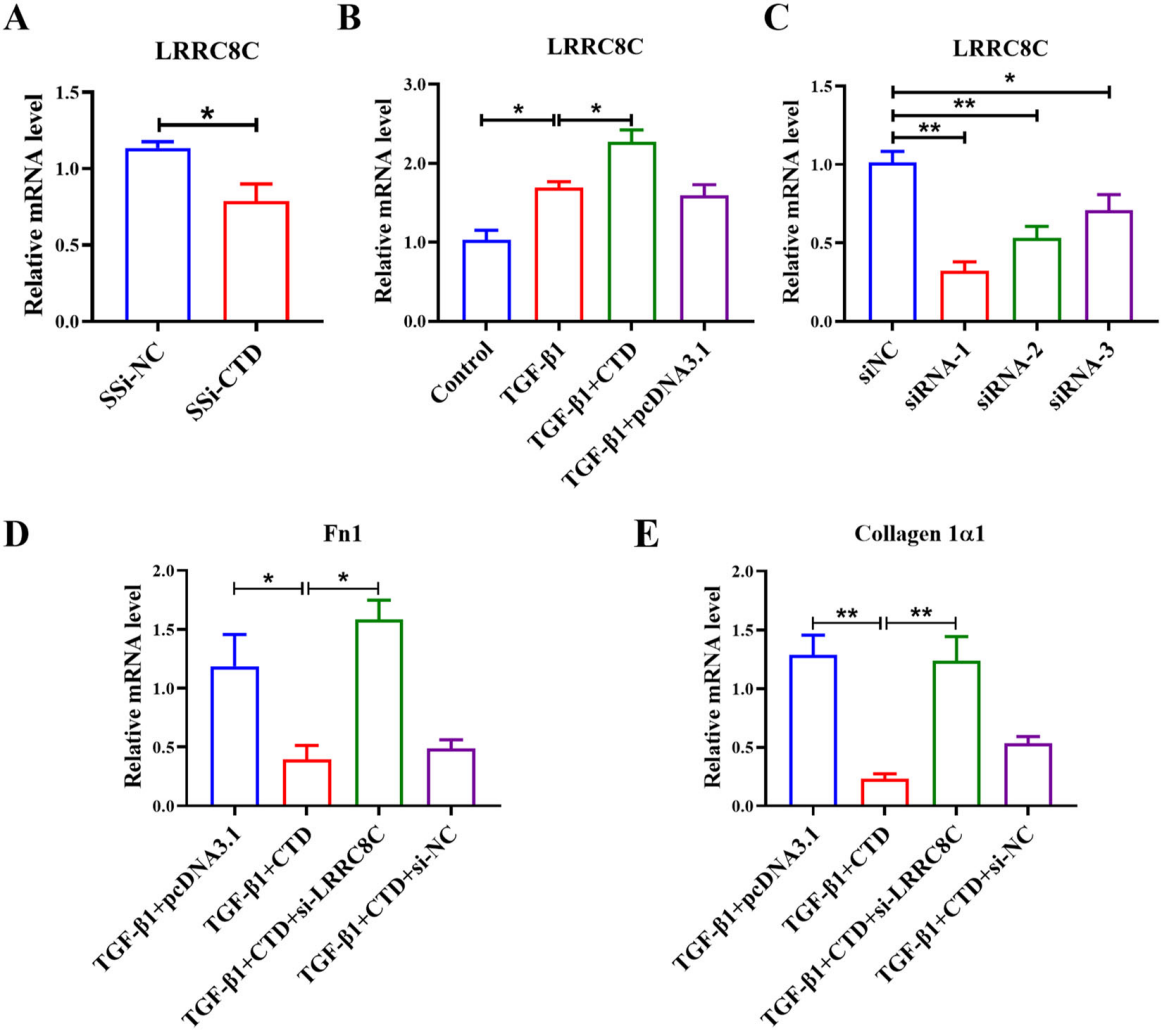
LncRNA *CTD-2528L19.6* regulates *LRRC8C* in MRC-5 cells. **A**, **B** qRT-PCR shows that silencing or forced overexpression of *CTD-2528L19.6* inhibited (**A**) or promoted (**B**) the expression of *LRRC8C*. **C-E** Silencing of *LRRC8C* restored the expression levels of Fn1 and Collagen 1α1 in TGF-β1-treated MRC-5 cells. *n* = 4; *P < 0.05, **P < 0.01.

In the co-expression network, expression of *CTD-2528L19.6* was positively correlated with *LRRC8C* in both early-stage (*r* = 0.95, *P* = 2.82E-4, Pearson correlation test, Fig. 3A) and advanced-stage IPF (*r* = 0.81, *P* = 8.71E-3, Pearson correlation test, Fig. 3D). Therefore, we performed qRT-PCR assay to examine the effect of *CTD-2528L19.6* on *LRRC8C*. Results showed that knockdown of *CTD-2528L19.6* inhibited the expression of *LRRC8C* in MRC-5 cells (Fig. 8A), whereas overexpression of *CTD-2528L19.6* promoted *LRRC8C* expression at mRNA level (Fig. 8B).

Then, we constructed three small interference RNAs (siRNAs) against *LRRC8C* to further explore the effects of *LRRC8C* knockdown on collagen deposition. qRT-PCR results showed that all three siRNAs could inhibit the expression of *LRRC8C* in MRC-5 cells (Fig. 8C). As shown in Fig. 8D, E, silencing of *LRRC8C* alleviated the inhibitory effect of *CTD-2528L19.6* on Fn1 and Collagen 1α1 in TGF-β1-treated MRC-5 cells.

## Discussion

In this study, by systematical analysis of transcriptome profile, we revealed a set of differentially expressed lncRNAs in IPF compared with normal lung tissues. Interestingly, some lncRNAs were up-regulated in the transition from normal lung to early-stage IPF, subsequently down-regulated during the transition to advanced-stage IPF. The lncRNA-mRNA co-expression network-based transcriptome analysis revealed a key fibrosis regulator lncRNA *CTD-2528L19.6* in IPF. We demonstrated that lncRNA *CTD-2528L19.6* participates in the pathogenesis and progression of IPF in different stages with different mechanisms. In MRC-5 cells, up-regulation of *CTD-2528L19.6* prevented the fibrosis progression of IPF by alleviating fibroblast activation. LncRNA *CTD-2528L19.6* inhibited fibroblast activation through regulating the expression of *LRRC8C* in vitro assays. Silencing of *CTD-2528L19.6* promoted fibroblast activation, which may be a novel biomarker for IPF.

Expression of *CTD-2528L19.6* was increased in early-stage of IPF and decreased in advanced-stage IPF. The dysregulation and effect of *CTD-2528L19.6* in IPF is similar to the *MMP13*, whose expression is up-regulated during two stages after fibrosis whereas knockdown of *MMP13* could aggravate the progression of lung fibrosis^34,35^. Thus, we inferred that, at the early-stage of IPF, the adaptive up-regulating *CTD-2528L19.6* is sufficiently to partially offset the activation of fibroblasts. However, with long-term stress, a sustained increase of the fibroblasts beyond a certain threshold could counteract the function of *CTD-2528L19.6* up-regulation, leading to the development of fibroblast activation. Also, the biological experiments in our work revealed that enhanced expression of lncRNA *CTD-2528L19.6* prevents the activation of fibroblast, which confirmed our hypothesis.

*CTD-2528L19.6* may prevent the progression of IPF and exhibit anti-fibrotic activity by regulating the expression of fibrosis associated mRNAs. In IPF pathogenesis network, mRNA *DCLRE1C* is a member of the non-homologous end joining pathways, and deleterious mutations which can cause severe combined immunodeficiency^36^. In early-stage IPF progression networks, mRNA *S100A8* has been reported in bronchoalveolar lavage fluid as a biomarker of IPF fibrosis^31^. In advanced-stage IPF progression network, mRNA *THBS1* is co-expressed with *CTD-2528L19.6*, which activates the reproduction of fibroblast cells in mice^37^. *TLR7* has been revealed to be a novel IPF associated gene and biomarker^32^, and is dysregulated in IPF pathogenesis^38^. In IPF progression network, genes *S100A8* and *TLR7*, which were positively correlated with the expression of *CTD-2528L19.6*, act as immune cell markers^32^. The crosstalk analysis between mRNAs and lncRNAs highlights the important role of immune regulation in IPF, which warrants further detail in vitro or in vivo biological experiments. The functional relationship between *LRRC8C* and *CTD-2528L19.6* in fibrosis was proved in vitro assays. LncRNA *CTD-2528L19.6* inhibited fibroblast activation through regulating the expression of *LRRC8C*. Limited by the sample amount of IPF data set, we did not control the FDR when performing differential expression analysis. However, the roles of *CTD-2528L19.6* in IPF predicted by bioinformatics analysis could be validated by cellular experiments and some correlated genes were proved to be involved in IPF by functional annotation. Another key lncRNA *NR2F1-AS1* with many transcripts was not tested in this study, which is worthy of our follow-up in-depth research. Notably, the lncRNA *TP53TG1*, which is most frequently co-expressed with mRNAs in IPF pathogenesis network, also deserves our following study.

Overall, we predicted IPF pathogenic and progression related lncRNAs and mRNAs through analyzing transcriptional profile, then constructed an IPF pathogenic lncRNA-mRNA co-expression network and dynamic IPF progression networks. A key lncRNA *CTD-2528L19.6* was captured to regulate the pathogenesis and progression of IPF as a potential biomarker. *CTD-2528L19.6* may regulate fibroblast activation in IPF progression by mediating the expression of some mRNAs, such as *LRRC8C, DDIT4, THBS1, S100A8* and *TLR7*. Our results suggest that during early-stage, *CTD-2528L19.6* was up-regulated to prevent IPF, while *CTD-2528L19.6* was down-regulated to promote fibroblast activation from early-stage to advanced-stage transition. This finding provides a framework for designing interventions that could prevent the development or progression of fibrosis at various stages of IPF development. The present research also reveals some lncRNAs as potential IPF biomarkers.

## Materials and methods

### Microarray data and processing

Gene expression dataset (GSE24206)^39^, with 17 IPF (8 early-stage IPF and 9 advanced-stage IPF) and 6 normal lung tissues, was downloaded from the Gene Expression Omnibus (GEO, http://www.ncbi.nlm.nih.gov/geo/) (Supplementary Fig. S1A). Expression for the Affymetrix U133 Plus 2.0 GeneChips was estimated by robust multi-array average then log2 transformed. Data were filtered prior to analysis: If a gene was mapped to multiple probe sets, the expression value for the gene was generated by averaging. Probes that did not map to any Gene ID or map to multiple Gene IDs were deleted^40^.

GSE10667 and GSE73854 were collected from the GEO and SRP10849 was download from The Sequence Read Archive (SRA, https://www.ncbi.nlm.nih.gov/sra/).

### Identification of IPF signature

We performed three comparisons using Student’s *t*-test: (1) early-stage IPF *vs*. normal; (2) advanced-stage IPF *vs*. normal; (3) advanced-stage IPF *vs*. early-stage IPF. We selected genes with *P* < 0.01 as DEGs, including DE lncRNAs and DE mRNAs. The DEGs derived from both (1) and (2) were defined as IPF pathogenic signatures. And, the DEGs detected from both (1) and (3) were defined as IPF progression signatures, as showed in Supplementary Fig. S1B.

### Co-expression network construction

Pearson correlation test was used to test the expression correlation between differential expressed lncRNAs and mRNAs. The lncRNA-mRNA pairs with the absolute value of correlation coefficient *r* > 0.8 and *P*-value < 0.01 were included in the co-expression network.

Then, a pathogenic co-expression network between IPF pathogenic lncRNAs and mRNAs was constructed in IPF samples. Two IPF progression co-expression networks between IPF progression lncRNAs and mRNAs were constructed in early-stage IPF and advanced-stage IPF samples, respectively (Supplementary Fig. S1C).

### Fibrosis related gene sets and IPF cell markers

Fibrosis related gene sets were collected from the Molecular Signatures Database (MSigDB, https://www.gsea-msigdb.org/gsea/msigdb), including fibroblast related gene sets. These annotated sets of genes involved in fibrosis related biochemical pathways, signaling cascades, expression profiles from research publications, and other biological concepts^41^. IPF cell markers (*KRT5*, *MUC5AC*, *MUC5B*, *NGFR*, *SCGB1A1*, *SPDEF*) were collected from the CellMarker database (http://biocc.hrbmu.edu.cn/CellMarker/)^42^.

### Pathway enrichment analysis

Pathway information was downloaded from Kyoto Encyclopedia of Genes and Genomes (KEGG) on August 17, 2017 (http://www.genome.jp/kegg/), consists of six top categories (09100 to 09160) for KEGG pathway maps. The hypergeometric distribution model was used to test whether KEGG pathways were enriched with genes in the co-expression network. The *P*-value was adjusted by the Benjamini-Hochberg (BH) procedure. The pathways with a false discovery rate (FDR) less than 0.05 were considered to be significant.

### MRC-5 cell culture and transfection

MRC-5 cell line was purchased from Cell Bank of Chinese Academy of Sciences, and cultured in 89% DMEM (Biological Industries, Israel) containing 10% FBS (Biological Industries, Cromwell, CT, USA) and 1% Penicillin-Streptomycin-Amphotericin B (Solarbio, China) then plated in a standard humidity incubator at 37 °C with 5% CO_2_. PCR was used to synthesize the full-length of lncRNA *CTD-2528L19.6* and inserted into the pcDNA3.1 vector. pcDNA3.1 empty vector was used as control. LncRNA smart silencer SSi-*CTD*/SSi-NC (negative control) was constructed by RiboBio Tech (Guangzhou, China). *CTD-2528L19.6* plasmid or SSi-*CTD*/SSi-NC and Lipofectamine 2000 (Invitrogen, Carlsbad, CA, U.S.A.) transfection reagents were mixed with Opti-MEM (GIBCO, Grand Island, NY, U.S.A.) in serum-free medium, respectively. Then incubated for 5 min and protected from the light. Mixed two liquids and placed for 15 min at room temperature, then added to the cell plate and cultured in incubator. After transfection 6 h replaced with a normal culture medium. After cells were cultured with 10 ng/ml TGF-β1 (Sigma-Aldrich, U.S.A.) for 48 h and prepared for further analysis (Supplementary Fig. S1D).

### Fluorescence in situ hybridization

Fluorescence in situ hybridization (FISH) was performed using the lncRNA FISH Probe Mix kit (Ribobio, Guangzhou, China). MRC-5 cells were fixed in 4% paraformaldehyde (Solarbio, China) for 10 min and then added 1 ml precooling permeable solution (5 μl Triton-X + 1 ml PBS) for 5 min at 4 °C. Added 200 μl pre-hybridization buffer into each well then incubated at 37 °C for 30 min. Then discarded the pre-hybridization buffer and added 150 μl hybridization buffer with lncRNA FISH Probe Mix, hybridized overnight at 37 °C. Later washed with buffer I (4x SSC), buffer II (2x SSC) and buffer III (1x SSC) at 42 °C. The nuclei were stained with DAPI (Roche Molecular Biochemicals, Basel, Switzerland). The images were taken under the inverted fluorescence microscope (Olympus, IX73, Japan).

### RNA extraction and quantitative RT-PCR

The total RNA of cells was extracted with TRIzol reagent. The concentration and purity of extracted RNA were determined by NanoDrop 8000 (Thermo, U.S.A). RNA reverse transcription for cDNA by using 5× All-in-One RT Master Mix. cDNA was used to detect the relative expression of mRNA by real-time quantitative reverse transcriptase polymerase chain reaction (qRT-PCR) in the presence of SYBR Green fluorescent dye (Applied Biosystems, Foster City, CA). The relative expression level was calculated based on the Ct values and GAPDH was used as a normalized control.

### Protein extraction and Western blot

Total proteins of MRC-5 cells were extracted and lysed with RIPA buffer (Beyotime, Jiangsu, China) containing protease inhibitor. Protein samples were separated on 8% sodium dodecyl sulfate-polyacrylamide gel and transferred to pure nitrocellulose (Pall Life Sciences, Ann Arbor, MI, USA) after electrophoresis. The membranes were probed with primary antibodies against β-actin (1:500, 66009-1-Ig, Proteintech, Wuhan, China), Fn1 (1:500, 15613-1-AP, Proteintech, Wuhan, China) and Collagen I (1:500, WL0088, Wanlei, Liaoning, China). The protein expression levels were detected by the Odyssey Infrared Imaging System (Odyssey CLX, Biosciences, USA).

### Immunofluorescence staining

After transfected and treated with TGF-β1, MRC-5 cells were fixed by 4% paraformaldehyde at room temperature for 30 min, and then permeabilized with 0.1% Triton X-100. Then, the cells were blocked with 50% goat serum and incubated with anti-Rabbit α-SMA antibody (1:100, ab7817, Abcam). Finally, cells were incubated with anti-rabbit IgG (H + L) (Alexa Fluor 488 Conjugate) (1:500, 4412, CST). Nuclei were stained with DAPI. Immunofluorescence images were photographed under fluorescence microscope.

### EdU cell proliferation assay

According to the Cell-Light EdU DNA cell proliferation kit (RiboBio, Guangzhou, China) instructions, the cells were incubated with 200 μl, 50 μM EdU solution. The cells were fixed with 4% paraformaldehyde and incubated in a 2 mg/ml glycine shaker. Afterwards, cells were stained with Apollo solution staining for proliferating cells. Nuclei were stained by DAPI. Images were finally photographed under the inverted fluorescence microscope.

### Wound-healing migration assay

Wound-healing migration assay was performed as previously described^24^. MRC-5 cells were seeded in 6-well plates and grown until formation of confluent monolayer. Then cells were gently scratched with a 10 μl pipette tip. The scratch healing areas were observed and photographed under microscope (×20 objective). Afterwards, the cells were transfected and added with TGF-β1. The images were taken by the Nikon Ts100 microscope (Nikon, Tokyo, Japan) and analyzed by using Image-J. First, the images resolution was changed to 8-bit and adjust the contrast of the enhanced image. After that, smoothed the scratch edge then found edge again. Finally, set the appropriate image threshold and measured the scratch area. Wound healing rate (%)= 1-(24 h or 48 h scratch area /0 h scratch area)*100.

### Statistics and analysis

Student’s *t*-test was used for statistical analysis between two groups. The fitting regression was constructed to modeling the relationship between *CTD-2528L19.6* and IPF cell markers with linear fitting using formula = y~x with method = “lm” in R package ggplot2. All bioinformatics analyses were carried out using R software version 3.6.0 (http://www.r-project.org/). Cytoscape software (version 3.6.0) was used to visualize the lncRNA-mRNA co-expression networks. All experimental data were presented as mean ± SEM. One-way analysis of variance (ANOVA) was used to determine the statistically significant differences among multiple groups. Statistical analyses were carried out using GraphPad Prism 8.0.

## Supplementary information

supplement file

## Data Availability

The datasets analyzed during the current study are available from the public databases.
