# Supplementary material for: LncRNA CTD-2528L19.6 prevents the progression of IPF by alleviating fibroblast activation
Source: Cell Death Dis. 2021 Jun 10;12(6):600. doi: 10.1038/s41419-021-03884-5 (PMC8192779; doi:10.1038/s41419-021-03884-5)
Supplement: Supplementary file 1 — supplement file [file 41419_2021_3884_MOESM1_ESM.pdf]

Supplementary Information for

LncRNA CTD-2528L19.6 prevents the progression of IPF by alleviating fibroblast activation

Tingting chen *et al.*

Fig. S1. Flowchart of this study.. 2

Fig. S2. Identification of IPF mRNA signatures... 3

Fig. S3. The expression of lncRNA *CTD−2528L19.6* in three independent datasets... 4

Fig. S4. Illustration of fibrosis related gene sets that contain the mRNAs co-expressed with lncRNAs in IPF... 5

Fig. S5. The expression of six cell markers in GSE24206. 6

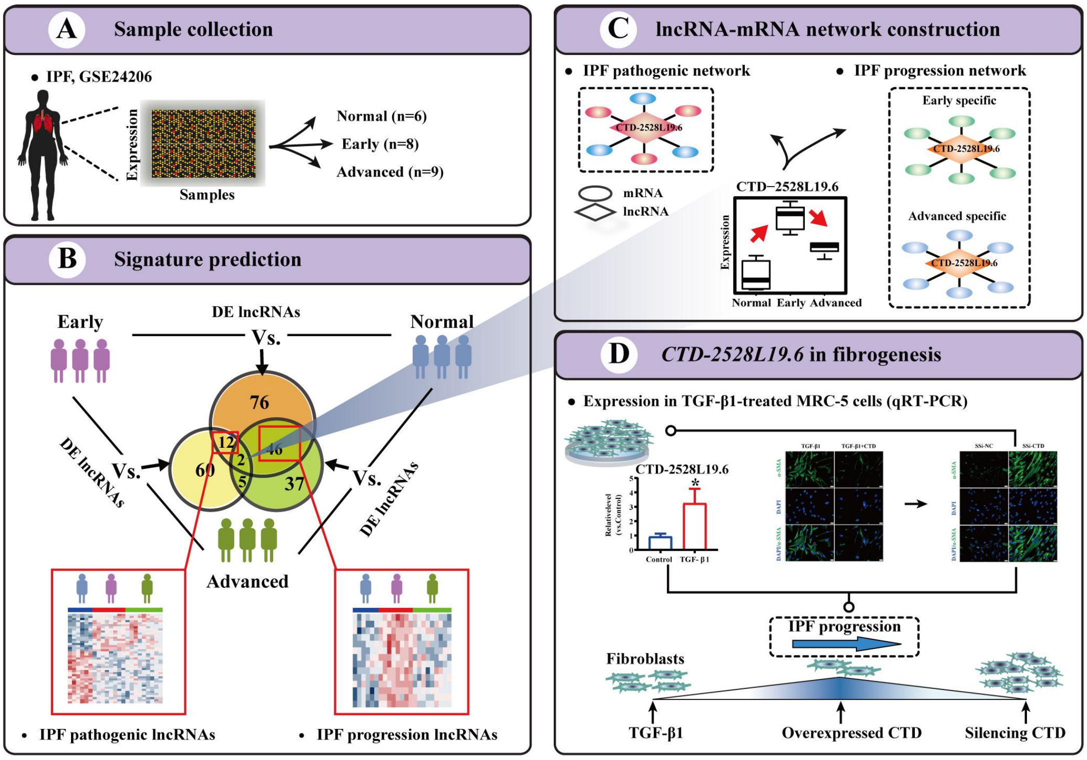

Fig. S1. Flowchart of this study.

(A) IPF data collection and preprocessing. (B) Identification of IPF pathogenic and progression signatures. (C) Construction of lncRNAs-mRNAs co-expression network, including IPF pathogenic network and IPF progression networks. (D) qRT-PCR and human lung fibroblast MRC-5 cells assays *in vitro* to determine the effect of lncRNA *CTD-2528L19.6* in lung fibrosis. DE lncRNAs, differentially expressed lncRNAs. CTD, *CTD2528L19.6.*

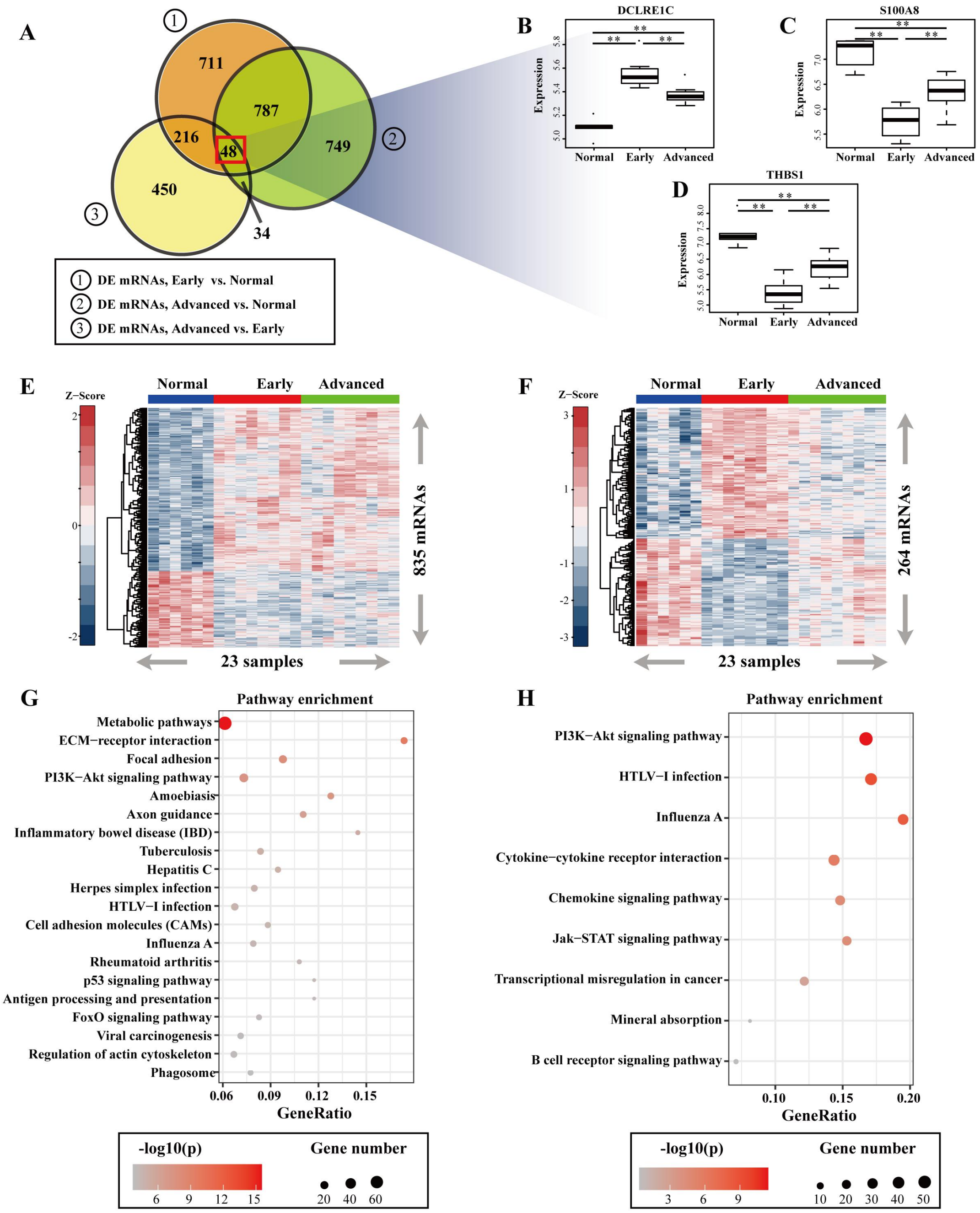

Fig. S2. Identification of IPF mRNA signatures.

(A) Venn diagram of overlapping DE lncRNAs among three groups. (B-D) Distribution of expression levels for three of the key mRNAs *DCLRE1C*, *S100A8* and *THBS1*, **P* < 0.05, ***P* < 0.01 in Student's t test. (E, F) Heatmaps show the global expression levels of IPF pathogenic mRNAs (E) and IPF progression mRNAs (F) in GSE24206. (G) Top 20 terms of significantly enriched KEGG pathways with IPF pathogenic mRNAs (*FDR* < 0.05, Hypergeometric test). (H) All KEGG pathways

were significantly enriched with IPF progression mRNAs (*FDR* < 0.05, Hypergeometric test). Size of bubble represents the gene number enriched in the pathway. Degree of correlation was depicted as degree of red. DE mRNAs, differentially expressed mRNAs.

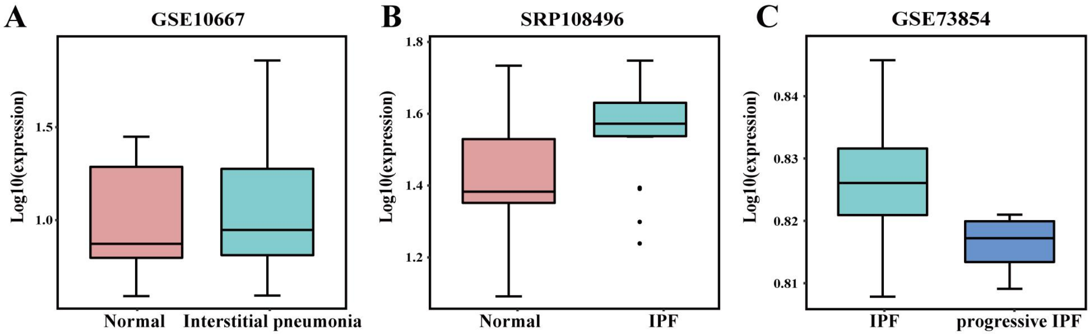

Fig. S3. The expression of lncRNA *CTD−2528L19.6* in three independent datasets. GSE10667 (A) (n = 15 : 31), SRP10849 (B) (n = 8 : 18) and GSE73854 (C) (n = 4 : 4).

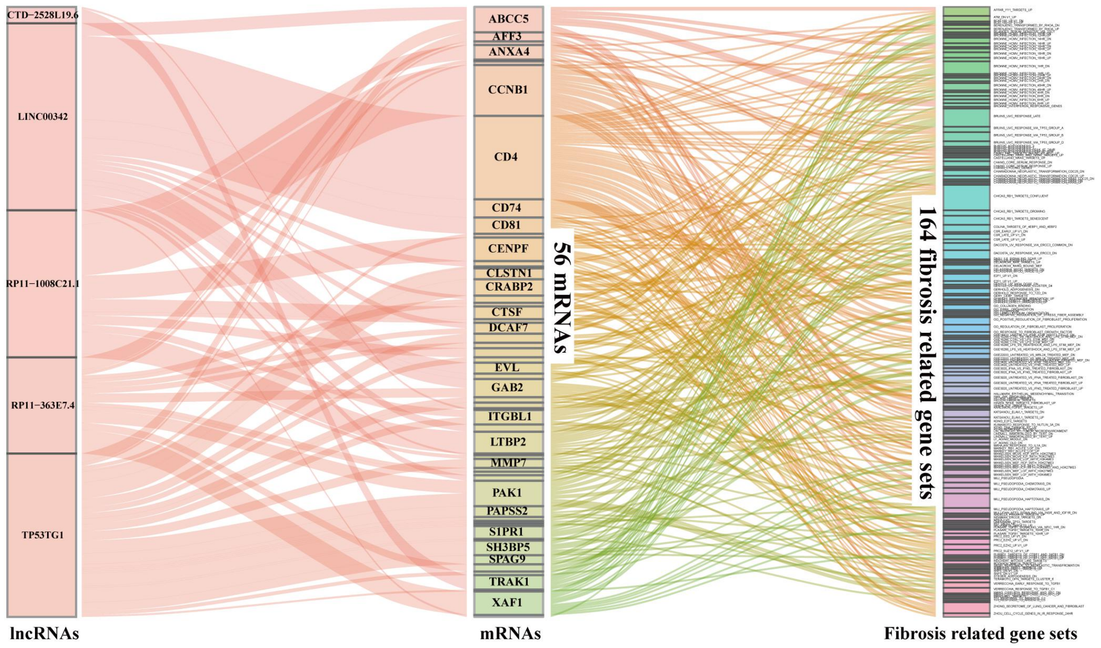

Fig. S4. Illustration of fibrosis related gene sets that contain the mRNAs co-expressed with lncRNAs in IPF. Sankey diagram displays the connections between lncRNAs and co-expressed mRNAs (| r | > 0.8, *P* < 0.01, Pearson correlation test) and fibrosis related gene sets in which the mRNAs participate in. For clarity, some mRNAs that rarely participate in fibrosis related gene sets were unlabeled.

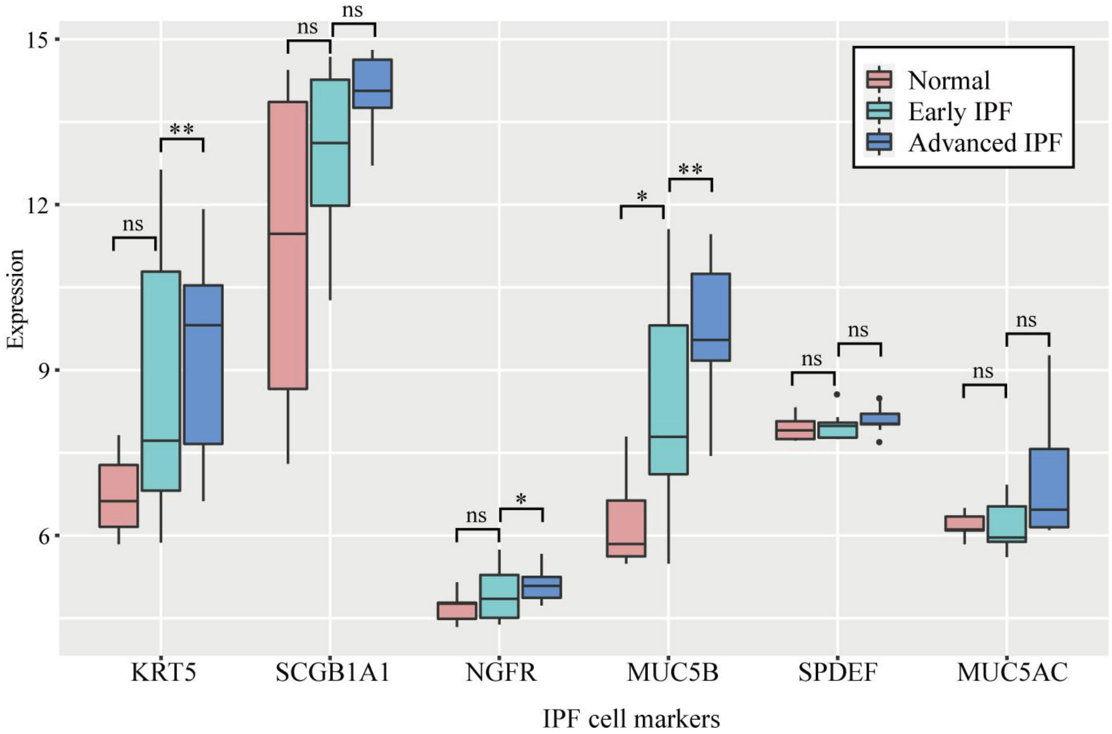

Fig. S5. The expression of six IPF cell markers in GSE24206. *P < 0.05, **P < 0.01 in Student's t test. "ns", not significance.
